# Production of the forskolin precursor 11*β*-hydroxy-manoyl oxide in yeast using surrogate enzymatic activities

**DOI:** 10.1186/s12934-016-0440-8

**Published:** 2016-02-26

**Authors:** Codruta Ignea, Efstathia Ioannou, Panagiota Georgantea, Fotini A. Trikka, Anastasia Athanasakoglou, Sofia Loupassaki, Vassilios Roussis, Antonios M. Makris, Sotirios C. Kampranis

**Affiliations:** Department of Biochemistry, School of Medicine, University of Crete, P.O. Box 2208, 71003 Heraklion, Greece; Department of Pharmacognosy and Chemistry of Natural Products, School of Pharmacy, University of Athens, Panepistimiopolis Zografou, 15771 Athens, Greece; Institute of Applied Biosciences – Centre for Research and Technology Hellas (INAB-CERTH), P.O. Box 60361, 57001 Thermi, Thessaloniki, Greece; Mediterranean Agronomic Institute of Chania, P.O. Box 85, 73100 Chania, Greece

**Keywords:** Cytochrome P450, Metabolic engineering, Natural products, Terpene, Isoprenoid

## Abstract

**Background:**

Several plant diterpenes have important biological properties. Among them, forskolin is a complex labdane-type diterpene whose biological activity stems from its ability to activate adenylyl cyclase and to elevate intracellular cAMP levels. As such, it is used in the control of blood pressure, in the protection from congestive heart failure, and in weight-loss supplements. Chemical synthesis of forskolin is challenging, and production of forskolin in engineered microbes could provide a sustainable source. To this end, we set out to establish a platform for the production of forskolin and related epoxy-labdanes in yeast.

**Results:**

Since the forskolin biosynthetic pathway has only been partially elucidated, and enzymes involved in terpene biosynthesis frequently exhibit relaxed substrate specificity, we explored the possibility of reconstructing missing steps of this pathway employing surrogate enzymes. Using CYP76AH24, a *Salvia pomifera* cytochrome P450 responsible for the oxidation of C-12 and C-11 of the abietane skeleton en route to carnosic acid, we were able to produce the forskolin precursor 11*β*-hydroxy-manoyl oxide in yeast. To improve 11*β*-hydroxy-manoyl oxide production, we undertook a chassis engineering effort involving the combination of three heterozygous yeast gene deletions (*mct1*/*MCT1*, *whi2*/*WHI2*, *gdh1*/*GDH1*) and obtained a 9.5-fold increase in 11*β*-hydroxy-manoyl oxide titers, reaching 21.2 mg L^−1^.

**Conclusions:**

In this study, we identify a surrogate enzyme for the specific and efficient hydroxylation of manoyl oxide at position C-11*β* and establish a platform that will facilitate the synthesis of a broad range of tricyclic (8,13)-epoxy-labdanes in yeast. This platform forms a basis for the heterologous production of forskolin and will facilitate the elucidation of subsequent steps of forskolin biosynthesis. In addition, this study highlights the usefulness of using surrogate enzymes for the production of intermediates of complex biosynthetic pathways. The combination of heterozygous deletions and the improved yeast strain reported here will provide a useful tool for the production of numerous other isoprenoids.

**Electronic supplementary material:**

The online version of this article (doi:10.1186/s12934-016-0440-8) contains supplementary material, which is available to authorized users.

## Background

**Fig. 1:**
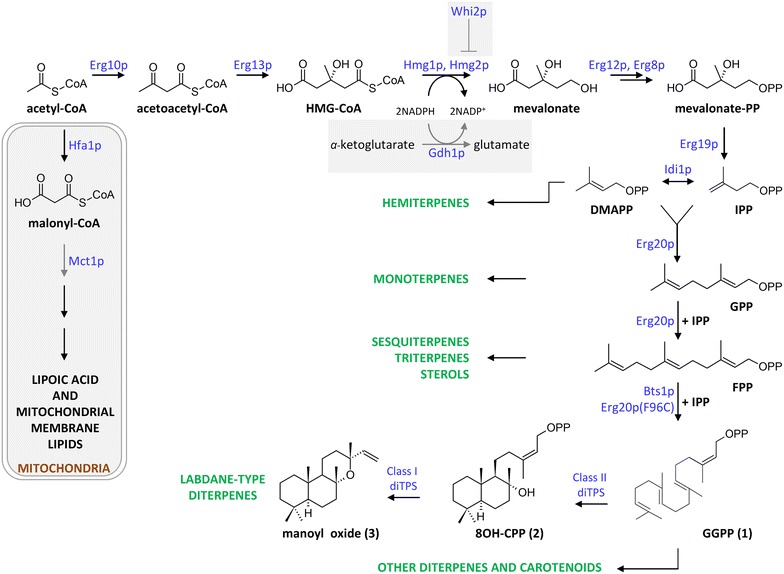
Schematic representation of isoprenoid biosynthesis via the mevalonic acid pathway. The mevalonate pathway provides the substrates for the different terpene classes [dimethylallyl diphosphate (DMAPP) for hemiterpenes (C_5_), geranyl diphosphate (GPP) for monoterpenes (C_10_), farnesyl diphosphate (FPP) for sterols, sesquiterpenes (C_15_) and triterpenes (C_30_), geranylgeranyl diphosphate (GGPP) for diterpenes (C_20_) and carotenoids (C_40_)]. Labdane-type diterpene biosynthesis from GGPP, via 8OH-CPP, is described using manoyl oxide as the example. Whi2p, previously identified as positive genetic interactor of HMG2 [38], and pathways competing for substrates involved in terpene biosynthesis, such as the mitochondrial fatty acid biosynthesis pathway or glutamate biosynthesis, are also indicated. Enzyme names correspond to the *S. cerevisiae* proteins. Steps downregulated in this study by heterozygous deletion of the corresponding gene are indicated by *gray arrows*

Plant natural products have found numerous applications in fragrances, flavors or pharmaceuticals, but the exploitation of many of these compounds is hampered by limited availability or seasonal variation of the raw materials. Reconstruction of natural product biosynthetic pathways in engineered microbes can provide a sustainable alternative and has so far been successful in providing access to important industrial compounds (e.g. artemisinin, resveratrol, vanillin [1–3]). Among the plant natural products with industrial application, terpenes (or isoprenoids) form the largest and more diverse group. Terpenes are synthesized by the successive addition of the 5-carbon isoprene building block, giving rise to classes of compounds with increasing skeletal size, such as hemiterpenes (C_5_), monoterpenes (C_10_), sesquiterpenes (C_15_), and diterpenes (C_20_) (Fig. 1).

**Fig. 2:**
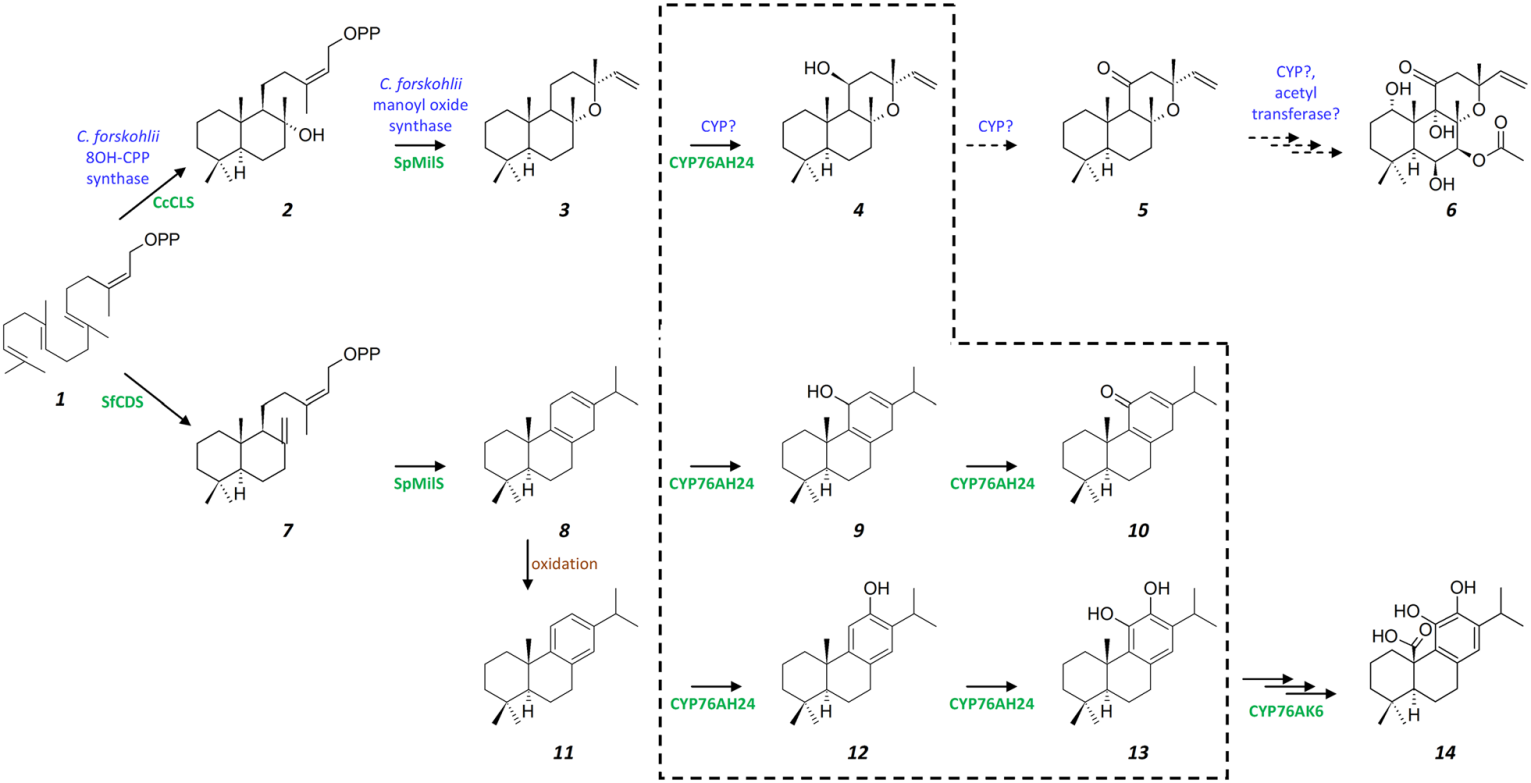
Proposed biosynthetic pathway of forskolin and carnosic acid. Both pathways begin from the common diterpene precursor GGPP (*1*). In *C. forskohlii*, a class II diTPS converts GGPP to 8OH-CPP (*2*), which is then taken up by a class I enzyme, to form manoyl oxide (*3*). This, in turn, becomes oxidized at several positions (C-1, C-6, C-7, C-11), presumably by the action of specific CYPs, and eventually O-acetylated at the C-7 hydroxy to generate forskolin (*6*). 11*β*-hydroxy-manoyl oxide (*4*) and 11-oxo-manoyl oxide (*5*) are believed to be the first steps in this mechanism, although the enzyme(s) catalyzing these reactions in *C. forskohlii* have not yet been identified. In *S. pomifera*, GGPP is converted to CPP (*7*) and then to miltiradiene (*8*) by corresponding class II (CPP synthase; CDS) and class I (Miltiradiene synthase; SpMilS) diTPSs. Miltiradiene is non-enzymatically converted to abietatriene (*11*), the substrate of CYP76AH24. CYP76AH24 catalyzes two successive oxidation events, one on C-12 of abietatriene producing ferruginol (*12*), and a second one on C-11 of ferruginol producing 11-hydroxy-ferruginol (*13*). When provided with miltiradiene, in vitro or in yeast cells, CYP76AH24 catalyzes a two step oxidation leading to 11-keto-miltiradiene (*10*), via 11-hydroxy-miltiradiene (*9*) [26]. The *dashed box* encloses the reactions catalyzed by CYP76AH24. CYP76AK6 takes up 11-hydroxy-ferruginol to catalyze a three step oxidation leading to carnosic acid (*14*) [26]. The promiscuous class I diTPSs, SpMilS, can also accept 8OH-CPP (*2*) to produce manoyl oxide (*3*) [25] To reconstruct the first steps of the forskolin biosynthetic pathway in yeast, CcCLS was used to produce 8OH-CPP, SpMilS was employed to convert 8OH-CPP to manoyl oxide, and CYP76AH24 was exploited to oxidize manoyl oxide (*3*) to 11*β*-hydroxy-manoyl oxide (*4*)

Within the diterpene class, the superfamily of labdane-related diterpenes comprises more than 7000 members and is characterized by a basic decalin core [4]. Their biosynthesis requires the cyclization of geranylgeranyl diphosphate (GGPP), by a class II diterpene synthase (diTPS), to a labdane-type diphosphate (e.g. (+)-copalyl diphosphate ((+)-CPP) or 8-hydroxy-CPP (8OH-CPP)), which is subsequently taken up by a class I diTPS to generate the basic diterpene skeleton (Fig. 1). Through the action of decorating enzymes, such as cytochrome P450s (CYPs), these basic labdane-type skeletons are converted to a diverse array of highly complex molecules [4]. Several labdane-type diterpenes, such as tanshinones, carnosic acid, and forskolin, display potent biological activities. Tanshinones are isolated from the roots of *Salvia miltiorrhiza* and exhibit strong antioxidant and anti-inflammatory activities, and cardiovascular and cerebrovascular therapeutic effects [5–8]. Carnosic acid (**14**) and carnosol are well known antioxidants produced in rosemary and *Salvia* spp., active as anti-adipogenic [9] and anticancer agents [10, 11]. Forskolin (**6**) is a complex heterocyclic labdane diterpene produced in the root cork cells of *Coleus forskohlii* plant [12]. By activating adenylyl cyclase, it increases the intracellular cAMP levels and acts as a positive inotropic agent and a vaso- and broncho-dilator [13–17]. Forskolin and its derivatives have found application in weight-loss supplements [18, 19], in the treatment of open angle glaucoma [20], in the control of blood pressure [21], and in the protection from congestive heart failure [22]. Enantioselective chemical synthesis of forskolin has not yet been successful, calling for the application of biotechnological methods for its production. However, the forskolin biosynthetic pathway has only been partially elucidated. The first steps are believed to involve the formation of (13*R*)-manoyl oxide (**3**) from GGPP (**1**), through 8OH-CPP (**2**), and the enzymes involved in these reactions have been identified and characterized [12]. The highly oxygenated structure of forskolin requires the oxidation of five different carbon atoms of the manoyl oxide skeleton and the acetylation of the resulting 7*β*-OH group. One of the early events in this process likely involve oxidation of manoyl oxide at position C-11, since 11-oxo-manoyl oxide (**5**) (Fig. 2) was isolated from *C. forskohlii* roots [23, 24], but a catalytic activity responsible for this reaction has not yet been identified.

We recently established a “plug-and-play” platform to facilitate the reconstruction of the diversity of terpenes in yeast [25]. This approach revealed that class I diTPSs and related CYPs frequently exhibit significant substrate promiscuity, a property that can be exploited to provide surrogate enzymatic activities for orphan biosynthetic steps. In the process of biosynthetic pathway elucidation, a Synthetic Biology approach taking advantage of surrogate enzymes for the production of putative precursors of molecules of interest can serve as a useful tool. Production of intermediates or precursors in microbial “chassis”, in titers that enable facile isolation and purification of these compounds, can yield complex substrates for activity screening using in vitro assays or coupled in vivo platforms, greatly speeding up the full process [26]. Here, we apply such an approach in the production of the forskolin precursor 11*β*-hydroxy-manoyl oxide, taking advantage of two promiscuous enzymes from *S. pomifera*: one class I diTPS that is normally responsible for the conversion of (+)-CPP to miltiradiene [27], a key intermediate in the biosynthesis of carnosic acid-related diterpenes, and one CYP targeting C-12 and C-11 of the abietatriene skeleton [26]. Combined with a set of heterozygous yeast gene deletions (*MCT1*, *WHI2*, *GDH1*) aiming to improve manoyl oxide availability, we achieved a fourfold increase in manoyl oxide conversion efficiency and a 9.5-fold increase in 11*β*-hydroxy-manoyl oxide titers, reaching 21.2 mg L^−1^. This approach sets the basis for the heterologous production of forskolin in yeast and provides a tool for the elucidation of subsequent steps of forskolin biosynthesis, such as hydroxylation at positions C-1, C-6, C-7 or C-9.

## Results and discussion

### Establishing manoyl oxide production in yeast using a surrogate terpene synthase

**Fig. 3:**
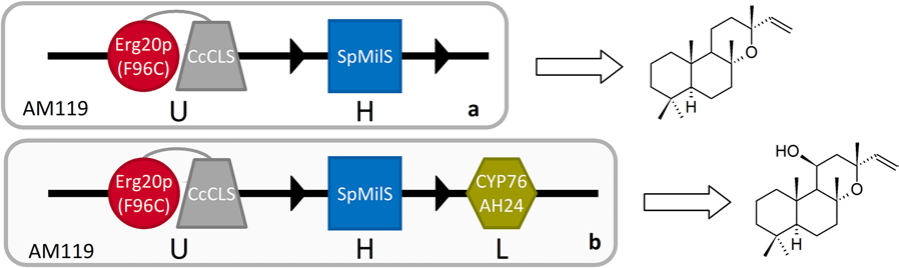
Diagrammatic illustration of configuration of the yeast platform for the production manoyl oxide and 11*β*-hydroxy-manoyl oxide. **a** The fusion between the class II diTPS, CcCLS, responsible for 8OH-CPP formation and the yeast variant Erg20p(F96C), engineered to synthesize GGPP, under uracil selection (U) was co-expressed in yeast cells with the class I diTPS, SpMilS, under histidine (H) selection, for the production of manoyl oxide. **b** Introduction of CYP76AH24 in the above system, under leucine (L) selection, enabled oxidation of manoyl oxide to 11*β*-hydroxy-manoyl oxide

Due to the so far limited knowledge of the final steps of its biosynthetic pathway, we set out to reconstruct forskolin biosynthesis in yeast using surrogate diTPS and CYP activities. In our previous work, we discovered that several class I diTPSs examined were able to accept alternative substrates and to yield different products with similar efficiency [25]. One such example was *S. pomifera* miltiradiene synthase (SpMilS), an enzyme believed to accept (+)-CPP as its physiological substrate to produce miltiradiene. Miltiradiene is a common precursor in the biosynthesis of tanshinones and carnosic acid-related diterpenes and several miltiradiene synthases have been reported in different organisms, including *S. miltiorrhiza,S. pomifera*, *S. fruticosa* and *Rosmarinus officinalis* [27–30]. When SpMilS is provided with the 8-hydroxy form of CPP, 8OH-CPP, it produces primarily manoyl oxide [25]. Taking advantage of this observation, we exploited SpMilS to set up an efficient manoyl oxide-producing yeast system in strain AM119 (Table 1). AM119 was selected because it presents a favorable chassis for the development of a modular platform that can incorporate additional oxidation steps. In yeast, overproduction of exogenous CYPs frequently leads to poor oxidation efficiency due to heme depletion [31]. Increasing the levels of the limiting enzyme of the heme biosynthetic pathway, encoded by the *HEM3* gene, was found to overcome heme depletion and to improve bioconversion [31]. Thus, to provide a chassis amenable to the incorporation of multiple CYP oxidation events, strain AM119 was developed by chromosomal integration of the *HEM3* gene under the control of the strong P_TDH3_ promoter in the 3′-UTR of the *FLO5* locus of strain AM102 [26]. In order to establish manoyl oxide production in AM119, in addition to SpMilS, the fusion between the *Cistus creticus* 8OH-CPP synthase (*Cc*CLS) and a GGPP-producing yeast Erg20p mutant (Erg20p(F96C)) was also expressed to provide high levels of the 8OH-CPP precursor (Fig. 3a). Under these conditions, the manoyl oxide titer at the end of shake-flask batch cultivation reached 41 mg L^−1^ of yeast culture. The reported titer for manoyl oxide production using the *C. forskolii* manoyl oxide synthase is 10 mg L^−1^ [33].

**Table 1:** List of *S. cerevisiae* strains used

Strain	Genotype	Source
AM119	Mat a/α, P_Gal1_-*HMG2*(K6R)::HOX2, *ura3*, *trp1*, *his3*, P_TDH3_-*HMG2*(K6R)*X*2-::*leu2 ERG9*/*erg9*, *ubc7*/*UBC7*, *ssm4*/*SSM4*, P_TDH3_-*HEM3*-*FLO5*	Ref. [26]
AM119-1	Mat a/α, P_Gal1_-*HMG2*(K6R)::HOX2, *ura3*, *trp1*, *his3*, P_TDH3_-*HMG2*(K6R)*X*2-::*leu2 ERG9*/*erg9*, *ubc7*/*UBC7*, *ssm4*/*SSM4*, P_TDH3_-*HEM3*-*FLO5*, P_TDH3_-*CcGGPPS1*-*FLO8*	This study
AM119-2	Mat a/α, P_Gal1_-*HMG2*(K6R)::HOX2, *ura3*, *trp1*, *his3*, P_TDH3_-*HMG2*(K6R)*X*2-::*leu2 ERG9*/*erg9*, *ubc7*/*UBC7*, *ssm4*/*SSM4*, *mct1*/*MCT1*, P_TDH3_-*HEM3*-*FLO5*, P_TDH3_-*CcGGPPS1*-*FLO8*	This study
AM119-3	Mat a/α, P_Gal1_-*HMG2*(K6R)::HOX2, *ura3*, *trp1*, *his3*, P_TDH3_-*HMG2*(K6R)*X*2-::*leu2 ERG9*/*erg9*, *ubc7*/*UBC7*, *ssm4*/*SSM4*, *mct1*/*MCT1*, *whi2*/*WHI2*, P_TDH3_-*HEM3*-*FLO5*, P_TDH3_-*CcGGPPS1*-*FLO8*	This study
AM119-4	Mat a/α, P_Gal1_-*HMG2*(K6R)::HOX2, *ura3*, *trp1*, *his3*, P_TDH3_-*HMG2*(K6R)*X*2-::*leu2 ERG9*/*erg9*, *ubc7*/*UBC7*, *ssm4*/*SSM4*, *mct1*/*MCT1*, *whi2*/*WHI2*, *gdh1*/*GDH1*, P_TDH3_-*HEM3*-*FLO5*, P_TDH3_-*CcGGPPS1*-*FLO8*	This study

### Oxidation of manoyl oxide by CYP76AH24

**Fig. 4:**
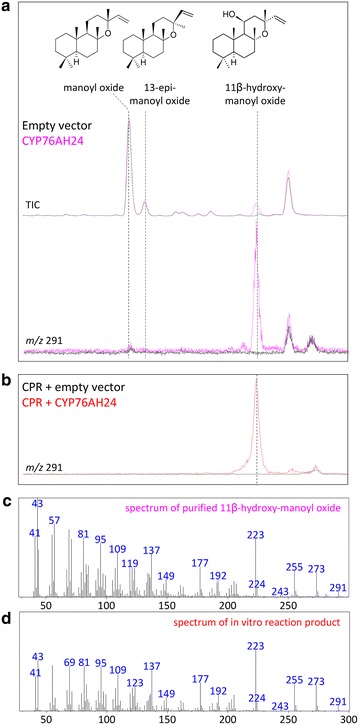
Formation of 11*β*-hydroxy-manoyl oxide by CYP76AH24. **a** Expression of CYP76AH24 in manoyl oxide-producing yeast cells resulted in the production of a new compound, which after isolation and structural analysis was identified as 11*β*-hydroxy-manoyl oxide. **b** GC–MS chromatogram of the products of an in vitro reaction using microsomal CYP76AH24 protein, manoyl oxide as substrate and NADPH as co-factor. A preparation of yeast microsomal membranes from a strain that expressed only CPR2 is used as control. Production of 11*β*-hydroxy-manoyl oxide was confirmed by comparison of retention time and mass spectrum with purified compound. **c** Mass spectrum of 11*β*-hydroxy-manoyl oxide standard (isolated from engineered yeast cells and characterized by NMR spectroscopy). **d** Mass spectrum of 11*β*-hydroxy-manoyl oxide produced by CYP76AH24 in an in vitro reaction

Recently, we discovered CYP76AH24 from *S. pomifera* as an enzyme responsible for the oxidation of C-12 of abietatriene to produce ferruginol and the subsequent oxidation of C-11 of ferruginol to yield 11-hydroxy-ferruginol (Fig. 2), two important intermediates in the carnosic acid biosynthetic pathway. When provided with miltiradiene, CYP76AH24 catalyzed two successive oxidation events on C-11, giving rise to 11-keto-miltiradiene (Fig. 2; [26]). Thus, CYP76AH24 appears to exhibit relatively relaxed substrate selectivity, being able to accept several structurally related molecules and to catalyze their oxidation at C-12 or C-11. Assuming that additional compounds with similar structure may also be oxidized by CYP76AH24, we decided to investigate its ability to catalyze conversion of manoyl oxide to related oxidized structures. Manoyl oxide-producing AM119 yeast cells were engineered to express CYP76AH24 and poplar CPR2 [33] from the bidirectional vector pESC-Leu under the P_GAL10_ and P_GAL1_ promoters, respectively (Fig. 3b). GC–MS analysis of the dodecane extracts of the corresponding cultures revealed one new peak compared to the empty vector control, likely corresponding to a manoyl oxide oxidation product (Fig. 4a). To examine whether additional manoyl oxide oxidation products are formed, which are either retained by yeast cells or inefficiently volatilized during GC–MS analysis, yeast cultures were extracted with pentane and the extracts were derivatized with Sylon-HTP (Sigma-Aldrich) prior to analysis. No additional peak was detected, suggesting that CYP76AH24 likely catalyzes the synthesis of one main oxidized form of manoyl oxide.

**Fig. 5:**
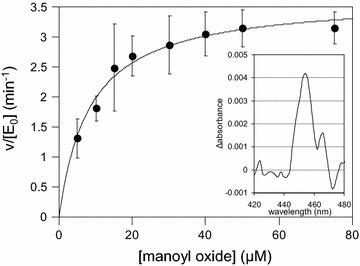
Steady-state kinetic analysis of the oxidation of manoyl oxide by CYP76AH24. The enzymatic activity of CYP76AH24 was evaluated using varying concentration (1–75 μM) of manoyl oxide substrate and 80 pmol of enzyme. The produced 11*β*-hydroxy-manoyl oxide was quantified by CG–MS analysis using purified compound as standard. Quantification of CYP76AH24 enzyme concentration was performed by measuring the binding of CO to the reduced form of the purified enzyme (450 nm peak), according to [47]. The differential spectrum of the CO-treated enzyme preparation is shown in the *inset*

In order to identify the structure of the new compound, a large scale yeast culture (1 L) producing the unknown hydroxy-manoyl oxide derivative was established and the dodecane extract was fractionated and analyzed as described in the “Methods” section. Analysis of its NMR spectra revealed the identity of the new product as 11*β*-hydroxy-manoyl oxide (Additional file 1: Figs. S1 and S2, Table S2). To confirm that 11*β*-hydroxy-manoyl oxide is indeed the product of the CYP76AH24-catalyzed reaction and not the result of an in vivo bioconversion event, the enzymatic activity of CYP76AH24 was analyzed in vitro using purified yeast microsomal fractions in the presence of manoyl oxide and NADPH co-factor. A single product with retention time and mass spectrum matching that of purified 11*β*-hydroxy-manoyl oxide was detected (Fig. 4b–d). Kinetic analysis using the yeast microsomal preparation of CYP76AH24 revealed that this reaction occurs with high efficiency compared to the oxidation of C-11 of miltiradiene or ferruginol by CYP76AH24 (*k*_cat_/*K*_M_ = 315 × 10^3^ min^−1^ M^−1^ for manoyl oxide vs. 133 × 10^3^ min^−1^ M^−1^ for ferruginol and 29.3 × 10^3^ min^−1^ M^−1^ for miltiradiene; Fig. 5; Table 2).

**Table 2:** CYP76AH24 kinetic parameters with different substrates

Substrate	*K*_M_ (μM)	*k*_cat_ (min^−1^)	*k*_cat_/*K*_M_ (×10^3^ min^−1^ M^−1^)	Source
Manoyl oxide	11.6 ± 1.06	3.65 ± 0.12	315	This study
Ferruginol	45.84 ± 9.37	6.11 ± 0.45	133	Ref. [26]
Miltiradiene	72.63 ± 43.19	1.97 ± 0.72	27.1	Ref. [26]

### Chassis engineering to improve manoyl oxide and 11*β*-hydroxy-manoyl oxide production

Titers of 11*β*-hydroxy-manoyl oxide produced by AM119 cells reached 2.3 mg L^−1^, indicating a 5.3 % efficiency of manoyl oxide conversion (as estimated by the ratio of 11*β*-hydroxy-manoyl oxide titer to the sum of manoyl oxide and 11*β*-hydroxy-manoyl oxide titres). One factor limiting the efficiency of manoyl oxide oxidation may be a relatively low intracellular concentration of manoyl oxide. The estimated Michaelis–Menten constant (*K*_M_ = 11.6 μM) for the oxidation of manoyl oxide by CYP76AH24 suggests that if the intracellular concentration of manoyl oxide is in the low micromolar range, the enzyme will not function at maximum efficiency. Thus, increasing the yield of manoyl oxide synthesis may help improving the efficiency of conversion. To this end, a metabolic engineering effort aiming to improve strain AM119 as a chassis for diterpene production was undertaken.

*Incorporation of a plant GGPP synthase*—Initially, we aimed to improve the endogenous GGPP pool by constitutive expression of the *C. creticus* GGPP synthase (CcGGPPS) from a chromosomally integrated copy. The mature form of *Cc*GGPPS was incorporated in the 3′-UTR *FLO8* locus of AM119 strain, under the control of the P_TDH3_ constitutive promoter, generating strain AM119-1. No significant difference in either manoyl oxide or 11*β*-hydroxy-manoyl oxide titers was observed, suggesting that the Erg20p mutant used for GGPP production is providing sufficient levels of GGPP for 8OH-CPP synthesis and that this step is not limiting the pathway.

**Fig. 6:**
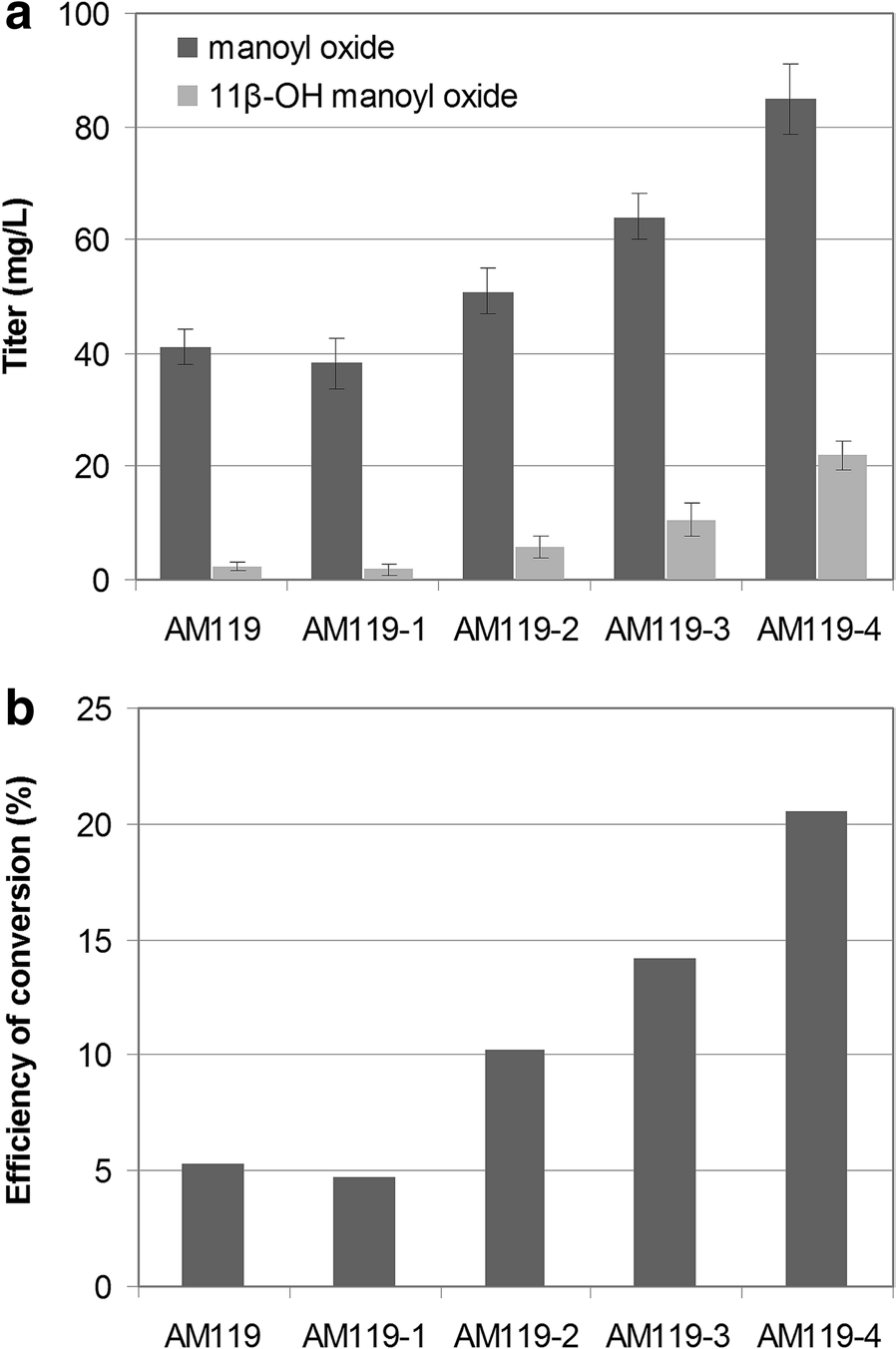
Optimization of 11*β*-hydroxy-manoyl oxide production in yeast cells. **a** Bar chart depicting the titers of manoyl oxide and 11*β*-hydroxy-manoyl oxide obtained using the different strains developed in this study. **b** Efficiency of conversion of manoyl oxide to 11*β*-hydroxy-manoyl oxide by CYP76AH24 in the different yeast strains developed. The efficiency of conversion is calculated as the ratio of 11*β*-hydroxy-manoyl oxide titer to the sum of manoyl oxide and 11*β*-hydroxy-manoyl oxide titres, expressed as percentage

*Heterozygous deletion of MCT1*—Isoprenoid biosynthesis in yeast proceeds mainly through the mevalonic acid pathway, which greatly relies on the availability of acetyl-CoA precursor. The acetyl-CoA pool is drained by competing pathways, such as ethanol production or membrane lipid and fatty acid biosynthesis, which divert acetyl-CoA from isoprenoid biosynthesis. One such pathway that competes for the availability of acetyl-CoA is the mitochondrial fatty acid biosynthesis pathway (or octanoyl-ACP pathway), which is responsible for the synthesis of lipoic acid and other mitochondrial membrane lipids [34]. This pathway is highly conserved and completely independent of the yeast cytosolic fatty acid synthase apparatus. In yeast, the second enzyme in this pathway is the malonyl-CoA:ACP transferase Mct1p (Fig. 1). Downregulation of *MCT1* is expected to redirect the acetyl-CoA substrate from fatty acid biosynthesis towards the mevalonate pathway and subsequently terpene biosynthesis. However, deletion of *MCT1* has been reported to result in a respiratory-deficient phenotype and small rudimentary mitochondria [35]. Deletion of one of the two alleles of a certain gene in a yeast diploid strain results in a decrease in the levels of the corresponding proteins by approximately 50 % [36]. To alleviate potential negative effects on cellular growth and viability caused by complete deletions, we previously reported the combination of monoallelic gene deletions as an efficient alternative which allowed the development of improved yeast strains for the production of the sesquiterpene *β*-caryophyllene [37]. Heterozygous deletion mutant yeast strains are stable and maintain their improved characteristic over time [37]. To downregulate the mitochondrial fatty acid synthase pathway without the deleterious effects of complete *MCT1* elimination, only one of the two *MCT1* alleles was deleted in strain AM119-1 to give rise to strain AM119-2 (Table 1). Heterozygous *MCT1* deletion improved 11*β*-hydroxy-manoyl oxide production by over twofold, reaching titers of 5.8 mg L^−1^ and boosted manoyl oxide conversion rate to 10.2 % (Fig. 6).

*Heterozygous deletion of WHI2*–Whi2p is a cytoplasmic scaffold protein required, together with its partner Psr1, for the proper activation of the general stress response. *WHI2* was identified as a positive genetic interactor of *HMG2* [38] and the heterozygous deletion *whi2*/*WHI2* was previously found to strongly synergize with *ubc7*/*UBC7* (already present in the AM119 background) in enhancing sesquiterpene production [37]. Heterozygous deletion of *WHI2* (*whi2*/*WHI2*) in the AM119-2 background generated strain AM119-3. In this chassis, 11*β*-hydroxy-manoyl oxide production increased by 83 % to 10.6 mg L^−1^, while manoyl oxide conversion reached 14.2 % (Fig. 6).

*GDH1 deletion*—NADP(+)-dependent glutamate dehydrogenase 1 (Gdh1p) synthesizes glutamate from ammonia and α-ketoglutarate (Fig. 1). A computational approach identified *GDH1* as a gene the deletion of which could improve carbon flux through the mevalonate pathway, by increasing the pool of NADPH available for Hmg1p and Hmg2p [39]. Deletion of *GDH1* in a haploid strain increased production of the sesquiterpene cubebol by 85 %. However, *GDH1* deletion also caused a significant decrease in the growth rate [39]. To avoid possible growth inhibition, a heterozygous *GDH1* deletion strain, AM119-4, was derived from AM119-3 (Table 1). When tested for 11*β*-hydroxy-manoyl oxide production, AM119-4 was 2 times more efficient than AM119-3, yielding 21.9 mg L^−1^ (Fig. 6), without any detectable growth retardation. The combination of the three heterozygous deletions (*mct1*/*MCT1*, *whi2*/*WHI2*, *gdh1*/*GDH1*) lead to an overall 9.5-fold increase of 11*β*-hydroxy-manoyl oxide titer over the base strain (AM119), and a fourfold improvement of manoyl oxide conversion (Fig. 6).

## Conclusions

Aiming to identify surrogate enzymes for the heterologous biosynthesis of forskolin, we identified CYP76AH24 as an enzyme that specifically catalyzes the introduction of one oxygen atom at the C-11*β* position of manoyl oxide to form the corresponding alcohol. An enzymatic activity capable of catalyzing this step is reported here for the first time. Further oxidation of 11*β*-hydroxy-manoyl oxide at the same position will generate the 11-oxo functionality present in forskolin. The efficiency (*k*_cat_/*K*_M_) of manoyl oxide oxidation by CYP76AH24 is high and comparative to the oxidation of C-11 of ferruginol, one of the presumed *in planta* substrates of CYP76AH24 (Table 2). The use of surrogate enzymes has also proved to be an efficient method for the production of manoyl oxide. The overall performance of the platform using SpMilS as the manoyl oxide synthase compares favorably to the published titers of manoyl oxide achieved using the corresponding *C. forskohlii* enzyme in *Escherichia coli* and in *Synechocystis* sp. PCC 6803 (40–100 vs. 10 mg L^−1^ in [33] and 0.46 mg L^−1^ in [40, 41], respectively).

Examination of the efficiency of the oxidation of manoyl oxide in yeast reveals that as the manoyl oxide titer increases, so does the efficiency of conversion of manoyl oxide to 11β-hydroxy-manoyl oxide (Fig. 6b). This suggests that at conditions where manoyl oxide production rates are low, most manoyl oxide does not accumulate long enough inside the cell (or in the ER) so as to be efficiently oxidized. This observation may provide important insights for future development of CYP-mediated oxidation of terpenes in yeast, as it indicates that efficient channeling of substrates between the terpene synthase step and the CYP-mediate oxidation is required.

By combining heterozygous deletions in three yeast genes we achieved significant yield improvements. This further supports the notion that monoallelic deletions can provide a useful genetic engineering tool in cases where complete gene deletion may have adverse effects in host cell physiology, as is the case here with *MCT1* and *GDH1*. Further fine-tuning of gene dosage could be achieved by complementary approaches, such as the integration of weaker promoters or destabilizing sequences in the mRNA [42].

The yeast platform described in this report opens the way for the elucidation of the biosynthesis of the unusual series of tricyclic (8,13)-epoxy-labdanes, providing either the starting material for in vitro reactions aiming to identify downstream steps of the pathway or a platform for the in vivo screening of candidate biosynthetic genes, as previously described for the elucidation of the carnosic acid pathway [26]. In addition, 11*β*-hydroxy-manoyl oxide produced by this platform can be further derivatized by chemical synthesis to yield analogues with potentially interesting properties. As already described for other terpenes [25], combinatorial biosynthesis coupled with protein engineering of labdane skeleton-acting CYPs can be applied to further derivatize the 11*β*-hydroxy-manoyl oxide molecule towards a broad range of potentially bioactive compounds.

## Methods

### Chemicals and enzymes

Standard compounds were obtained from: geranyllinalool (Aldrich, 48809), geranylgeraniol (Sigma, G3278), sclareol (VIORYL SA. Athens, Greece). Manoyl oxide standard was from our in-house collection, isolated from natural sources and characterized by NMR spectroscopy. PCR amplifications were performed using Phusion High-Fidelity DNA Polymerase (New England BioLabs, M0530) and MyTaq DNA polymerase (BIO-21105, Bioline). Restriction enzymes from New England BioLabs were used for cloning purposes. NucleoSpin Plasmid Kit (740588, Macherey–Nagel) was used for plasmid DNA purification, while QIAquick Gel Extraction Kit (#28704, Qiagen) was used for gel extraction and DNA purification.

### Yeast media, expression vectors, and strains

Yeast cells were cultivated in Complete Minimal (CM) medium, composed of 0.13 % (w/v) dropout powder (all essential amino acids), 0.67 % (w/v) Yeast Nitrogen Base w/o AA (Y2025, US Biologicals) and 2 % d-(+)-glucose monohydrate (16301, Sigma). For galactose-based medium, glucose was substituted with 2 % d-(+) galactose (G0625, Sigma) and 1 % Raffinose pentahydrate (R1030, US Biological). Constructs pYES2myc/CcCLS-ERG20(F96), pHTDH3myc/SpMilS and pESC-Leu::CPR2-CYP76AH24 were previously described [25, 26, 43].

The generation of yeast strain AM119 (Mat a/α, P_Gal1_-*HMG2*(K6R)::HOX2, *ura3*, *trp1*, *his3*, P_TDH3_-*HMG2*(K6R)*X*2-::*leu2 ERG9*/*erg9*, *ubc7*/*UBC7*, *ssm4*/*SSM4*, P_TDH3_-*HEM3*-*FLO5*) has previously been described [26]. AM119 was used as the starting strain to generate strain AM119-1. Plasmid construct COD7/CcGGDPS (PTDH3-CcGGDPS-CYC1t, LoxP-HIS5-LoxP) [37], which harbors the mature form of *C. creticus* GGPPS, was PCR amplified using primers 5-FLO8-COD7 and 3-FLO8-COD7 (Additional file 1: Table S1) incorporating flanking sequences complementary to the 3′UTR of *FLO8* gene. Following transformation, selection and excision of the selection marker, strain AM119-1 was generated.

The pUG27 cassette [44] was PCR amplified with primers MCT1-pUGF and MCT1-pUGR and used to transform AM119-1 cells to inactive one allele of *MCT1*. Proper integration of the cassette was verified by PCR on genomic DNA of selected colonies using primers MCT1prom and MCT1pUGR. Upon excision of the selection marker, strain AM119-2 was generated.

To generate strain AM119-3, the pUG27 cassette was PCR amplified with primers WHI2-640-pUGF and WHI2-2790-pUGR and was used to transform AM119-2 cells to inactive one allele of *WHI2*. Proper integration of the cassette was verified by PCR on genomic DNA of selected colonies using primers WHI2prom and WHI2-2790-pUGR, followed by selection marker excision.

Strain AM119-3 was subsequently transformed with a pUG27 PCR amplified cassette with primers GDH1-F-646-pUG and GDH1-R-2653 and to inactive one allele of *GDH.* Proper integration of the cassette was verified by PCR on genomic DNA of selected colonies using primers GDH1prom and GDH1-R-2653 pUG, and the selection marker was excised to give rise to strain AM119-4.

### Yeast strain cultivation, terpene quantification and extraction from yeast cells

Cultivation of yeast cells for the production of terpene compounds was performed as previously described [25]. For the expression of genes placed under the galactose-inducible promoters P_GAL1_ and P_GAL10_ (the CcCLS-Erg20p(F96C) fusion, poplar CPR2 and CYP76AH24), yeast cultures were grown until OD_600_ = 0.7–1 and subsequently switched to galactose-raffinose based selective growth medium (10–25 mL). Terpene extraction was performed by dodecane overlay (10 %) or liquid–liquid extraction using aliquots of 1 ml cultures and pentane as extracting solvent. Where appropriate, pentane extracts were derivatized using Sylon HTP (hexamethyldisilylazane:trimethylchlorosilane:pyridine, 3:1:9) (Supleco, Bellafonte, PA) as previously described [26]. GC-FID was employed for quantification and identification of terpene products as described in [45]. Identification of produced compounds by GC–MS analysis was carried out by comparison with commercial or in-house standards.

### Microsomal protein preparation and cytochrome P450 quantification

Yeast cultures (250 mL) were used to isolate microsomes from cells engineered to express CYP76AH24 with the method in [46], with an additional final ultracentrifugation step at 100,000*g* for 60 min. The concentration of CYP76AH24 was determined by the spectroscopic difference at 450 nm of CYP enzymes due to CO binding [47], using the extinction coefficient 91 mM^−1^ cm^−1^. Background correction of the endogenous CYPs was deduced using microsomes purified from cells carrying an empty vector.

### In vitro enzymatic assay and kinetic analysis

The enzymatic activities of CYP76AH24 was evaluated as previously described [25] using varying concentrations (1–75 μM) of manoyl oxide as substrate. The enzymatic reactions were incubated with shaking at 30 °C for 30 min and terminated by extraction with 100 μL of decane containg 10 μg/mL sclareol as internal standard. 2 μL of extracts were analyzed by GC–MS using the conditions previously described [45]. All assays were carried out in duplicates.

### General experimental procedures relating to the isolation and structure elucidation of 11*β*-hydroxy-manoyl oxide

NMR spectra were recorded on Bruker AC 200 and Bruker DRX 400 spectrometers. Chemical shifts are given on a *δ* (ppm) scale using TMS as internal standard. The 2D experiments were performed using standard Bruker pulse sequences. Low resolution EI mass spectra were measured on either a Hewlett-Packard 5973 mass spectrometer or a Thermo Electron Corporation DSQ mass spectrometer by using a Direct-Exposure Probe. GC–MS analyses were carried out using a Hewlett-Packard 6890 gas chromatograph equipped with a HP-5MS fused silica capillary column (30 m × 0.25 mm; film thickness 0.25 μm), a split-splitless injector and a Hewlett-Packard 5973 MS detector operating in electron ionization mode at 70 eV. Column chromatography separations were performed with Kieselgel 60 (Merck). HPLC separations were conducted using an Agilent 1100 Series liquid chromatography pump equipped with refractive index detector, using a Supelcosil LCSI Semiprep 5 μm (250 × 10 mm i.d.; Supelco) column. TLC were performed with Kieselgel 60 F254 (Merck aluminum support plates) and spots were detected after spraying with 15 % H_2_SO_4_ in MeOH reagent and heating at 100 °C for 1 min.

### Isolation of oxidation products

A 1 L culture of AM119 cells expressing the suitable plasmid in Gal/Raff-CM medium was overlaid with 100 mL of dodecane and incubated in a shake-flask for 2 days. The resulting dodecane layer was distilled in vacuo at 42 °C to afford a concentrated extract (ca. 5 mL) that was submitted to gravity column chromatography using *n*-pentane as the mobile phase in order to remove the remaining volume of dodecane. The column was flushed with EtOAc to retrieve the secondary metabolites. The solvent was evaporated in vacuo to yield an oily residue (1.07 g) which was submitted to gravity column chromatography on silica gel, using cyclohexane with increasing amounts of EtOAc as the mobile phase, to yield 6 fractions (1–6). Fraction 2 (104.0 mg) was subjected to normal phase HPLC, using cyclohexane/EtOAc (93:7) as eluent, to afford a subfraction (41.2 mg) that was further purified by normal phase HPLC, using *n*-Hex/EtOAc (94:6) as eluent, to yield 11*β*-hydroxy-manoyl oxide (**4**) (17.5 mg).

### Structure elucidation of isolated compounds

11*β*-Hydroxy-manoyl oxide (**4**) was identified on the basis of its spectroscopic data and comparison with literature values [48].

## Additional file

10.1186/s12934-016-0440-8 List of primers. **Table S2.** Experimental and bibliographic ^1^H and ^13^C NMR data (in CDCl_3_) of 11*β*-hydroxy-manoyl oxide (4). **Figure S1.** ^1^H NMR spectrum of 11*β*-hydroxy-manoyl oxide (4). **Figure S2.** ^13^C NMR spectrum of 11*β*-hydroxy-manoyl oxide (4).

## Abbreviations

CYP: cytochrome P450
GGPP: geranylgeranyl diphosphate
diTPS: diterpene synthase
CPP: copalyl diphosphate
8OH-CPP: 8-hydroxy-(+)-copalyl diphosphate
CcGGPPS: *Cistus creticus* geranylgeranyl diphosphate synthase
SpMilS: *Salvia pomifera* miltiradiene synthase
CcCLS: *Cistus creticus* 8-hydroxy-copalyl diphosphate synthase
CPR2: *Populus trichocarpa* × *Populus deltoides* cytochrome P450 reductase 2
NMR: nuclear magnetic resonance
CM medium: complete minimal medium
ACP: acyl carrier protein
