# Supplementary material for: Production of the forskolin precursor 11β-hydroxy-manoyl oxide in yeast using surrogate enzymatic activities
Source: Microb Cell Fact. 2016 Feb 26;15:46. doi: 10.1186/s12934-016-0440-8 (PMC4769550; doi:10.1186/s12934-016-0440-8)
Supplement: Supplementary file 1 — 10.1186/s12934-016-0440-8 List of primers. Table S2. Experimental and bibliographic 1H and 13C NMR data (in CDCl3) of 11β-hydroxy-manoyl oxide (4). Figure S1. 1H NMR spectrum of 11β-hydroxy-manoyl oxide (4). Figure S2. 13C NMR spectrum of 11β-hydroxy-manoyl oxide (4). [file 12934_2016_440_MOESM1_ESM.doc]

**Supplementary information**

Production of the forskolin precursor 11*β*-hydroxy-manoyl oxide in yeast using surrogate enzymatic activities

[Codruta Ignea](http://www.ncbi.nlm.nih.gov/pubmed/?term=Ignea C%5Bauth%5D)^1^, Efstathia Ioannou^2^, Panagiota Georgantea^2^, [Fotini A Trikka](http://www.ncbi.nlm.nih.gov/pubmed/?term=Trikka FA%5Bauth%5D)^3^, Anastasia Athanasakoglou^1^, Sofia Loupassaki^4^, Vassilios Roussis^2^, [Antonios M. Makris](http://www.ncbi.nlm.nih.gov/pubmed/?term=Makris AM%5Bauth%5D)^3*^ and [Sotirios C. Kampranis](http://www.ncbi.nlm.nih.gov/pubmed/?term=Kampranis SC%5Bauth%5D)^1*^

^1^Department of Biochemistry, School of Medicine, University of Crete, P.O. Box 2208, Heraklion 71003, Greece

^2^Department of Pharmacognosy and Chemistry of Natural Products, School of Pharmacy, University of Athens, Panepistimiopolis Zografou, Athens 15771, Greece

^3^Institute of Applied Biosciences – Centre for Research and Technology Hellas (INAB-CERTH), P.O. Box 60361, Thermi 57001, Thessaloniki, Greece

^4^Mediterranean Agronomic Institute of Chania, P.O. Box 85, Chania 73100, Greece

**Supplementary Tables**

**Table S1. List of primers**

| **Primer** | **Sequence** |
| --- | --- |
| 5-FLO8-COD7 | aatttggggatgggtttaagccctgtgaactgaaccattttcacagcaacgactcagttcgagtttatcattatc |
| 3-FLO8 -COD7 | accattatgccaagctacttcaatgagtgtacatcaaccagaaaagtgcctgtggatctgatatcaccta |
| MCT1-pUGF | acgtacactgagggtagtaacaaagcgttttgcacttttctgatcgtggtcagctgaagcttcgtacgc |
| MCT1-pUGR | caatgaatgcgtagaagcacagactgggagtggagagagttcgcagttttggcataggccactagtggatctg |
| MCT1prom | aatgcagaaagtgtcatcttcttacatgg |
| WHI2-640-pUGF | ttacagcataggcatagtgatagagtgtgagagagtctctaactcggcgtcacagctgaagcttcgtacgc |
| WHI2-2790-pUGR | accggatcaatgctgcttttgagtgatatttttcgaacaatgtacgactttattagcataggccactagtggatctg |
| WHI2prom | tcttatgctctagagtaataattactaagga |
| GDH1-F-646-pUG | tagtacgaccccacgtccaatcagcagagagaagctgttatcagctgctgcgccgcagctgaagcttcgtacgc |
| GDH1-R-2653 pUG | ggacgagtaaggtcatcaataagcctggtgtccaatcgatgcttacatacatagcataggccactagtggatctg |
| GDH1prom | tgaccatctgattggatggcctcga |

**Table S2. Experimental and bibliographic ^1^H and ^13^C NMR data (in CDCl_3_) of 11*β*-hydroxy-manoyl oxide (4)**

|  | **Experimental data** | | | **Bibliographic data^a^** | | |
| --- | --- | --- | --- | --- | --- | --- |
| **Position** | ***δ*_C_** | | ***δ*_H_ (*J* in Hz)** | ***δ*_C_** | | ***δ*_H_ (*J* in Hz)** |
| 1 | 39.3, | CH_2_ | 0.95 ddd (12.8, 12.8, 3.8), 1.73 m | 39.22, | CH_2_ | 0.95 ddd, 1.74 dt |
| 2 | 18.4, | CH_2_ | 1.37 m, 1.64 m | 18.42, | CH_2_ | 1.38 m, 1.64 tt |
| 3 | 41.9, | CH_2_ | 1.06 m, 1.34 m | 41.92, | CH_2_ | 1.07 ddd, 1.34 m |
| 4 | 33.2, | C |  | 33.19, | C |  |
| 5 | 57.0, | CH | 0.81 m | 57.03, | CH | 0.81 dd |
| 6 | 20.1, | CH_2_ | 1.31 m, 1.57 m | 20.14, | CH_2_ | 1.31 m, 1.58 m |
| 7 | 44.6, | CH_2_ | 1.41 m, 1.72 m | 44.56, | CH_2_ | 1.41 m, 1.73 dd |
| 8 | 74.8, | C |  | 74.80, | C |  |
| 9 | 56.5, | CH | 1.26 d (3.8) | 56.45, | CH | 1.26 d |
| 10 | 37.8, | C |  | 37.76, | C |  |
| 11 | 65.3, | CH | 4.40 ddd (6.0, 5.4, 3.8) | 65.24, | CH | 4.38 ddd |
| 12 | 44.2, | CH_2_ | 1.79 dd (14.2, 5.4), 1.93 dd (14.2, 6.0) | 44.20, | CH_2_ | 1.79 dd, 1.98 dd |
| 13 | 72.4, | C |  | 72.38, | C |  |
| 14 | 147.8, | CH | 5.80 dd (17.4, 10.7) | 147.77, | CH | 5.79 dd |
| 15 | 110.4, | CH_2_ | 5.07 dd (17.4, 1.4), 4.86 dd (10.7, 1.4) | 110.47, | CH_2_ | 5.07 dd, 4.86 dd |
| 16 | 29.8, | CH_3_ | 1.37 s | 29.73, | CH_3_ | 1.35 s |
| 17 | 27.4, | CH_3_ | 1.54 s | 27.44, | CH_3_ | 1.53 s |
| 18 | 33.5, | CH_3_ | 0.79 s | 33.50, | CH_3_ | 0.79 s |
| 19 | 21.4, | CH_3_ | 0.76 s | 21.38, | CH_3_ | 0.76 s |
| 20 | 17.1, | CH_3_ | 1.10 s | 17.12, | CH_3_ | 1.11 s |

^a^Topcu G, Tan N, Ulubelen A, Sun D, Watson WH: **Terpenoids and flavonoids from the aerial parts of Salvia candidissima**. *Phytochemistry* 1995, **40**(2):501-504.

**Supplementary Figures**

**Figure S1.** ^1^H NMR spectrum of 11*β*-hydroxy-manoyl oxide (**4**).

**Figure S2.** ^13^C NMR spectrum of 11*β*-hydroxy-manoyl oxide (**4**).
